# Skull thickness in skeletal deep bite: A predictive model for orthodontic treatment planning

**DOI:** 10.6026/973206300214488

**Published:** 2025-12-15

**Authors:** Siddharth Sonwane, Shweta Sonwane, Monica Chaurasia, Tanupriya Nigam

**Affiliations:** 1 Department of Orthodontics and Dentofacial Orthopaedics, Mansarovar Dental College, Bhopal, Madhya Pradesh, India; 2 Department of Oral Surgery, Government Dental College, Nagpur, Maharashtra, India; 3 Department of Dentistry, Government Medical College, Satna, Madhya Pradesh, India

**Keywords:** Skull thickness, skeletal deep bite, predictive model, orthodontic planning, cephalometry

## Abstract

Skull thickness plays a significant role in orthodontic diagnosis and treatment planning, particularly in skeletal malocclusions such as deep bite. In this retrospective cohort study, lateral cephalograms of 68 skeletal deep bite patients and 34 controls were assessed to measure frontal, parietal and occipital bone thickness and regression analysis was applied to establish predictive markers. Deep bite patients exhibited significantly greater parietal bone thickness than controls (p <0.05), with females showing higher parietal values and regression modeling identified parietal thickness as the most reliable predictor of skeletal deep bite (R^2^ = 0.61, p <0.01). These findings highlight that cranial bone thickness, particularly in the parietal region, may serve as a clinically useful predictor of skeletal deep bite and assist in orthodontic and surgical treatment planning.

## Background:

Craniofacial morphology plays a critical role in orthodontic diagnosis and treatment planning, particularly in vertical discrepancies such as skeletal deep bite, where altered mandibular rotation and reduced interbasal angles contribute to functional and esthetic concerns. Structural adaptations in the cranial vault have been reported in association with skeletal malocclusions, suggesting a potential link between cranial bone morphology and vertical dysplasia [1, 2]. Cranial thickness varies widely with age, sex and population differences, as demonstrated in anthropological and forensic studies [5, 6, 10 and 11]. Genetic influences, including variation in RUNX2 and other osteogenic regulators and functional factors such as occlusal loading further contribute to regional bone adaptation [7, 8]. Recent imaging-based research reinforces the relevance of cranial morphology in orthodontics. Three-dimensional CT studies show that skeletal configurations are associated with measurable differences in midface and cranial base geometry [16, 17]. Dolci *et al.* demonstrated that skull thickness and cranial geometry have biomechanical significance, supporting their use in analytical and clinical modeling [18]. Advances in cephalometric methodology also highlight the limitations of traditional two-dimensional radiography and emphasize the superior accuracy of 3D approaches for assessing craniofacial structures [19]. Moreover, current cephalometric investigations illustrate that vertical growth patterns and facial height ratios are closely related to skeletal divergence, a key feature of deep bite malocclusion [20]. Earlier work by Jacobsen *et al.* and Arntsen *et al.* reported increased parietal and occipital bone thickness in deep bite patients, with notable gender-related differences [1, 2]. However, contemporary studies exploring skull thickness as a diagnostic or predictive marker remain limited. Given the increasing evidence linking cranial morphology with skeletal growth patterns, evaluating skull thickness in skeletal deep bite may provide clinically relevant insights. Therefore, it is of interest to compare regional skull thickness between skeletal deep bite subjects and controls and to construct a predictive model to assist orthodontic planning.

## Methodology:

This retrospective cohort study evaluated skull thickness in individuals with skeletal deep bite compared with control subjects. Ethical approval was obtained from the Institutional Review Board (EC/13-11-2023/MDC/BLP-174). The sample included 68 adults with skeletal deep bite and 34 age-matched controls exhibiting neutral occlusion and normal vertical jaw relationships. Eligibility criteria included ages 18-31 years, presence of at least 24 permanent teeth and absence of prior orthodontic treatment, craniofacial anomalies, or systemic conditions affecting bone morphology. Standardized lateral cephalograms were obtained using a film-to-focus distance of 180 cm. Skull thickness was measured at predetermined frontal, parietal and occipital landmarks following established cephalometric protocols [4, 12]. To assess measurement reliability, 46 radiographs were randomly re-evaluated after two weeks, yielding intraclass correlation coefficients consistent with recommended standards [15]. Data distribution was examined using the Shapiro-Wilk test. Intergroup comparisons and sex-related differences were analyzed using unpaired t-tests, with significance set at p <0.05. Stepwise multiple regression analysis was performed to identify skull thickness parameters predictive of skeletal deep bite. All measurements and statistical procedures were conducted by calibrated investigators to minimize bias. The methodological approach allowed systematic evaluation of regional cranial thickness and its potential diagnostic relevance in skeletal deep bite.

## Results:

Regional skull thickness measurements demonstrated consistent anatomical patterns, with the parietal bone presenting the greatest thickness and the frontal bone the least across all participants. Individuals with skeletal deep bite exhibited significantly higher parietal and occipital thickness compared with controls (p <0.05), whereas frontal bone thickness showed no significant intergroup difference. Sex-specific analysis revealed that females had significantly greater parietal bone thickness than males (p <0.05), while no meaningful differences were observed for frontal or occipital regions. Stepwise regression analysis identified parietal bone thickness as the strongest predictor of skeletal deep bite (R^2^ = 0.61, p <0.01). Inclusion of occipital thickness provided only a minor improvement in predictive accuracy and frontal thickness did not contribute to the model. The overall findings indicate that increased parietal thickness is the most reliable cranial marker associated with skeletal deep bite (Table 1 - see PDF).

## Discussion:

The present study demonstrated that individuals with skeletal deep bite exhibit significantly greater parietal and occipital bone thickness than controls, while frontal thickness remains comparable. Parietal thickness emerged as the most robust predictor of skeletal deep bite in the regression model, indicating that cranial vault morphology may be closely linked to vertical dysplasia. These findings support earlier reports by Jacobsen and Arntsen, who observed increased cranial thickness in deep bite and other malocclusion groups [1, 2] and are consistent with anthropological data showing marked regional variation in cranial vault thickness [5, 6, 9, 10-11]. Sex-specific analysis revealed higher parietal thickness in females, aligning with previous observations of gender-related differences in cranial dimensions and remodeling patterns [3, 5, 9, 10-11]. Such variability likely reflects the combined effects of genetic determinants, including osteogenic regulators such as RUNX2 and functional adaptation to occlusal loading and masticatory forces [7, 8]. Recent three-dimensional imaging studies strengthen the biological plausibility of these associations. CT- and CBCT-based evaluations have shown that skeletal patterns are accompanied by measurable differences in midface morphology and cranial base dimensions [16, 17]. Sangeeth *et al.* further emphasized the biomechanical relevance of skull geometry, suggesting that cranial thickness may influence stress distribution and simulation outcomes [18]. Together with contemporary evidence linking vertical facial height ratios to growth patterns and skeletal divergence [20], the present results indicate that cranial vault thickness could represent an additional structural marker in patients with deep bite. From a clinical perspective, increased parietal and occipital thickness may have implications for planning skeletal anchorage and cranial bone harvesting, complementing previous work on parietal and occipital donor sites [13, 16, 17-18]. However, these applications require direct validation in surgical and anchorage-focused studies. Methodologically, the use of standardized lateral cephalograms ensured reproducibility and acceptable reliability [4, 12 and 15], but two-dimensional imaging cannot fully capture three-dimensional cranial morphology. Systematic reviews highlight the superior accuracy of 3D cephalometric approaches and support their use in future cranial thickness research [19]. Limitations also include the retrospective design, single-center sample and absence of longitudinal follow-up. Prospective multicenter studies using CT or CBCT, incorporating vertical growth pattern analyses and diverse populations, are needed to confirm and refine the predictive value of skull thickness in skeletal deep bite.

## Conclusion:

Individuals with skeletal deep bite exhibit significantly greater parietal and occipital bone thickness, with parietal thickness emerging as the strongest predictor of the malocclusion pattern. Data shows that cranial vault morphology may contribute to vertical dysplasia, with sex-related differences reflecting underlying biological variation in cranial bone adaptation. While skull thickness measurements may enhance diagnostic precision and treatment planning, further validation using three-dimensional imaging and larger cohorts is required to establish their predictive value in orthodontic and surgical applications.
